# Six-Year Environmental Surface Hygiene Monitoring in Hungarian School Kitchens (2019–2024): Hotspots, Seasonality, and One Health Implications

**DOI:** 10.3390/antibiotics15020120

**Published:** 2026-01-26

**Authors:** András Bittsánszky, Lili A. Lukács, Márton Battay, Miklós Süth, András J. Tóth

**Affiliations:** 1 Department of Food Hygiene, Institute of Food Chain Science, University of Veterinary Medicine Budapest, István u. 2., 1078 Budapest, Hungary; bittsanszky.andras@univet.hu (A.B.); toth.andras.jozsef@univet.hu (A.J.T.); 2 InDeRe Institute for Food System Research and Innovation Nonprofit Public Benefit Ltd., Budaörsi út 15. 1/11, 1118 Budapest, Hungary; 3 Department of Exotic Animal and Wildlife Medicine, University of Veterinary Medicine, István u. 2., 1078 Budapest, Hungary; battay.marton@univet.hu; 4 National Laboratory of Infectious Animal Diseases, Antimicrobial Resistance, Veterinary Public Health and Food Chain Safety, University of Veterinary Medicine Budapest, István u. 2., 1078 Budapest, Hungary

**Keywords:** institutional catering, school kitchens, food-contact surfaces, environmental monitoring, surface microbiota, antimicrobial resistance, biocides, One Health, microbiological risk assessment

## Abstract

**Background/Objectives**: Institutional catering serves vulnerable populations, including schoolchildren. Surfaces in food preparation environments are key control points for food safety and reservoirs and transmission routes for antimicrobial-resistant (AMR) bacteria. This study characterized the hygienic status of food-contact surfaces (FCS) and non-food-contact surfaces (NFCS) in Hungarian school kitchens, identified contamination hotspots, and examined how routine monitoring can support AMR prevention. **Methods:** We retrospectively analyzed routine environmental hygiene monitoring records from 96 school kitchens (2019–2024). In total, 8412 swab samples were collected, 8407 had quantifiable counts, 6233 from FCS (e.g., plates, trays, boards, utensils), and 2174 from NFCS (e.g., sinks, fridges, workers’ hands). Total aerobic mesophilic counts were measured with a redox-potential method and expressed as CFU/100 cm^2^; 250 CFU/100 cm^2^ (2.4 log_10_) was the hygienic threshold. **Results:** Overall, 12.4% of surfaces exceeded the threshold. Non-food-contact surfaces were more likely to be non-compliant than food-contact surfaces (OR 2.77, 95% CI 2.43–3.17; *p* < 0.001). Hotspots included transport-container lids (67.2% non-compliant; OR 43.82), sink basins (32.8%; OR 10.46), and cutting boards (21.6%; OR 5.89). Seasonally, non-compliance was highest in summer (16.5%) and lowest in winter (9.0%; *p* < 0.001). **Conclusions:** Multi-year monitoring revealed substantial contamination concentrated in a few hotspots that, within a One Health framework—which recognizes the interconnectedness of human, animal, and environmental health—may represent environmental reservoirs and cross-contamination nodes relevant to AMR prevention. Targeted optimization of cleaning and disinfection for these surfaces, combined with trend analysis of indicator data and periodic AMR-focused environmental sampling, could reduce foodborne and AMR-related risks in public catering.

## 1. Introduction

School catering affects the health of hundreds of thousands of children daily in Hungary; therefore, the food safety level of public catering is of paramount public health importance. Children’s immune systems are more vulnerable, infections can be more severe, and antibiotics administered during medical treatment exert further pressure on the development and spread of antimicrobial resistance (AMR). Recent One Health-oriented literature highlights that AMR is not only a hospital or human medical problem: the food chain, livestock farming, the environment, and the immediate surroundings of food production are also important resistance reservoirs [1,2]. At the intersection of microbial food safety and AMR are pathogens such as *Salmonella*, *Campylobacter*, *Listeria*, and ESBL-producing *Enterobacterales*, whose resistance poses an increasing challenge at all levels of the food chain [3]. Similarly, Fernández-Traporte et al. describe the “resistome” of the entire food chain, pointing out that resistance genes and resistant bacteria are present from farm to fork, and food-contact surfaces serve as important transmission nodes [4].

The fight against AMR can only be effective if human, animal, and environmental exposures are examined in an integrated manner, including “seemingly banal” spaces such as school kitchens and collective catering units. The presence of resistant pathogens in the kitchen environment can not only trigger acute foodborne infections but also contribute to the long-term circulation of resistance genes within the community. Accordingly, in a One Health perspective, food preparation environments form an interface between humans, foods of animal/plant origin, and downstream environmental pathways (e.g., wastewater); therefore, hygiene monitoring of kitchen surfaces can inform integrated prevention strategies [5].

### 1.1. Surface Hygiene and AMR—International Experiences

Numerous studies have detected significant microbial loads on kitchen surfaces, especially in high-traffic or collective catering environments. In Moroccan collective catering units, 17 different food-contact surfaces were examined, and several surface types—such as cutting boards and work surfaces—were found to have mesophilic total counts and hygiene indicators (*Enterobacteriaceae*, *S. aureus*) exceeding the limit [6]. Fallahizadeh et al. demonstrated in a restaurant environment that ATP and microbiological results of surfaces directly influence the outcome of official health inspections and can even lead to the temporary closure of units [7]. Viana et al. investigated surface contamination and resident behaviour in university student kitchens, finding that deficiencies in cleaning routines and inadequate hand hygiene were closely associated with higher surface counts, particularly in the sink area, on sponges, and on work surfaces [8]. These results align well with a previous survey in Hungarian school kitchens, where critical surfaces were identified using total aerobic counts, and the practical applicability of MicroTester-based rapid measurements was confirmed [9].

While most of these studies “only” measure general microbial load, increasing data are available showing that surfaces—especially wet, biofilm-covered, hard-to-clean zones—often harbour antibiotic-resistant strains and resistance genes as well [3,4,10,11].

### 1.2. Biocides, Surfaces, and Resistance

Disinfectants are used daily in public catering; however, there is growing evidence that the improper (under-dosed, too frequent, or poorly rinsed) application of biocides can contribute to the development of AMR. Widespread and often unjustified biocide use—especially in household and food industry environments—can promote the development of mechanisms (efflux pumps, cell membrane changes) that also strengthen cross- or co-resistance to antibiotics [12].

Investigating biocide resistance in *Klebsiella pneumoniae*, it was shown that adaptation to disinfectants often activates the same efflux systems or membrane changes that are also key in the development of antibiotic resistance [13]. O’Reilly et al. emphasize the challenges of laboratory testing for biocide susceptibility and the complexity of adaptation associated with long-term, low-dose exposure. This literature background is particularly relevant for the present study, as the school kitchen surfaces found to be critical (sink basins, food waste containers, transport container lids) are routinely and intensively disinfected—often with concentrations and contact times that are difficult to control—which could potentially favour the selection of biocide-tolerant and, in some cases, antibiotic-resistant strains [14].

### 1.3. Institutional Catering in Hungary and Rationale for the Study

In Hungary, the food safety of public catering is prescribed by EU regulations [15,16,17], national laws [18,19], and mandatory HACCP systems (based on GHP—Good Hygienic Practices). In practice, however, several domestic studies have confirmed that the effectiveness of documented self-monitoring systems strongly depends on the knowledge, attitude, and quality of real, daily practice of the workers [16,17,18,19,20,21]. An intervention study by Tóth et al. showed that a 20–30% improvement in storage, dishwashing, and cleaning practices can be achieved through targeted hygiene education [22]. At the same time, Illés et al. also demonstrated that the formal existence of documented HACCP systems alone does not guarantee low surface counts: surface MicroTester measurements showed a significant proportion of results above the limit [9]. Based on all this, it was justified to conduct a large-sample, multi-year study based specifically on surface microbiological data, which provides an objective picture of the hygienic status of school kitchens, reveals temporal and seasonal trends, identifies the most critical surfaces, and points out how surface hygiene is linked to reducing AMR risks.

### 1.4. Objectives

The main objective of the research is to numerically characterize the hygienic status of surfaces based on total mesophilic counts (CFU/100 cm^2^) using surface microbiological data collected in 96 Hungarian school catering kitchens between 2019 and 2024; to identify the food-contact (FCS) and non-food-contact (NFCS) surfaces that are most critical from a hygienic point of view; to investigate temporal (annual) and seasonal trends; and to interpret the results in terms of antimicrobial resistance (AMR) using literature on the relationship between surface microbiota and AMR.

### 1.5. Hypotheses

We hypothesized that (H1) non-compliance differs substantially across surface categories, with wet and hard-to-clean surfaces acting as hotspots, and (H2) hygiene performance showing seasonality.

## 2. Results

### 2.1. General Prevalence of Non-Compliant Surfaces

**Figure 1:**
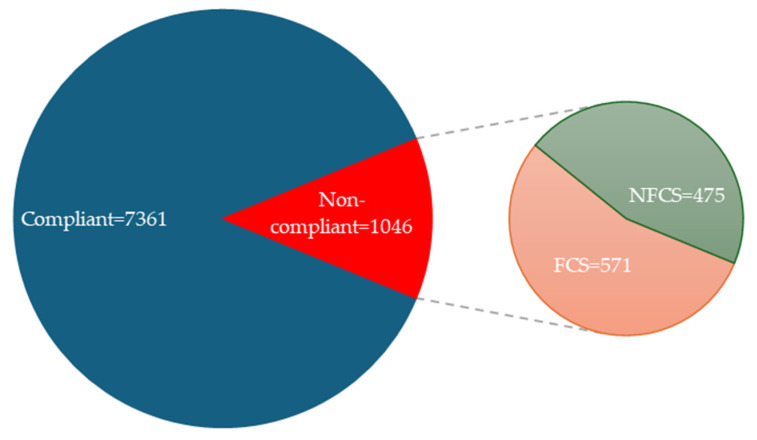
Sampling distribution (N = 8407) by compliance status and surface type (FCS vs. NFCS).

A total of 8412 environmental surface swabs were collected from institutional kitchens. Quantitative total mesophilic aerobic counts (estimated by a redox-potential method and expressed as CFU/100 cm^2^) results were available for 8407 samples; five samples were excluded from statistical analysis due to missing colony count data. Based on the 250 CFU/100 cm^2^ hygiene threshold, 12.4% of the samples exceeded the limit and were classified as non-compliant. Overall, 6233 samples were taken from food-contact surfaces (FCS), while 2174 samples were taken from non-food-contact surfaces (NFCS). In the FCS group, 571/6233 (9.2%) samples exceeded the threshold, while in the NFCS group, 475/2174 (21.8%) were non-compliant. The distribution of all samples above the limit between FCS and NFCS is shown in Figure 1. The 2 × 2 Chi-square test confirmed that non-compliance was significantly more frequent on NFCS than on FCS (χ^2^ = 237.0, *p* < 0.001). In a binary logistic regression model with non-compliance as the dependent variable and FCS/NFCS status as the predictor, the odds ratio (OR) for NFCS was 2.77 (95% CI: 2.43–3.17, *p* < 0.001) compared to FCS, indicating that NFCS were nearly three times more likely to exceed the hygiene threshold.

### 2.2. Differences Between Surface Categories

For the analysis by surface type, surface categories with at least 30 samples (n ≥ 30) were considered, resulting in 13 categories: plates, drinking cups/glasses, eating utensils, trays, work surfaces (table), refrigerator contact points, sink basins, food handlers’ hands, food waste containers, cutting boards, transport container lids, kitchen equipment, and jugs. The proportion of non-compliant samples differed significantly between surface categories (Table 1).

The chi-square test examining the relationship between surface category and non-compliance was highly significant (χ^2^ = 729.6, *p* < 0.001), indicating that the probability of exceeding the threshold was highly dependent on the type of surface. To quantify these differences, a logistic regression model was fitted with surface category as a categorical predictor, using the Plates category as a reference. The model reported a highly significant overall effect of surface type (likelihood ratio test *p* < 0.001). In the drinking cups, jugs, and general kitchen equipment categories, the odds of non-compliance did not differ significantly from the plates category (OR values were 1.26, 1.48, and 1.05, respectively; all *p* ≥ 0.22). Overall, these results highlight transport container lids, sink basins, cutting boards, food waste containers, handlers’ hands, and trays as the most critical surfaces from a hygienic point of view, where the odds of non-compliance were several-fold or even orders of magnitude higher than for plates.

### 2.3. Seasonal Variation in Hygienic Performance

Analyzed at the sample level, the proportion of non-compliant surfaces varied by season (Table 2).

The relationship between season and non-compliance was statistically significant (χ^2^ = 43.8, *p* < 0.001). In the logistic regression model using summer as a reference, the odds of non-compliance were significantly lower in all other seasons. Accordingly, the probability of surfaces exceeding the hygiene limit was highest in summer, medium in spring and autumn, and lowest in winter. To avoid increasing the sample size by treating each surface as independent, data were also aggregated at the kitchen-day level. For each kitchen and sampling day, the percentage of non-compliant surfaces was calculated, resulting in 880 kitchen-day observations.

One-way analysis of variance (ANOVA) showed a significant seasonal effect on the daily non-compliance rate (F(3, 876) = 7.85, *p* < 0.001). Average (±SD) non-compliance per kitchen day is shown in Table 3.

Tukey post hoc comparisons showed that non-compliance on summer days was significantly higher than in spring and winter, and that non-compliance in winter was significantly lower than in autumn. These kitchen-level results confirm the findings of the sample-level analysis and prove a clear, robust seasonal effect, with the worst hygienic performance during the summer period.

### 2.4. Year-to-Year Variations

The level of non-compliance also varied significantly between the studied years (2019–2024). The proportion of samples above the threshold per year was as follows (Table 4).

The Year × Non-compliance contingency table was significant (χ^2^ = 123.2, *p* < 0.001), suggesting that hygienic performance fluctuated over time and did not follow a simple linear trend. In a logistic regression model examining the effect of year as a categorical predictor (reference: 2019), the following results were obtained:

These results show that hygienic performance improved temporarily in 2021–2022 (lower odds of non-compliance compared to the 2019 level), followed by a marked deterioration in 2023, when the odds of non-compliance increased by approximately 55% relative to 2019. In 2024, the odds of non-compliance returned to a level similar to 2019.

### 2.5. Summary of Main Findings

Based on more than eight thousand surface swabs, approximately every eighth sample exceeded the 250 CFU/100 cm^2^ hygiene threshold. For non-food-contact surfaces (NFCS), the probability of non-compliance was nearly three times higher than for food-contact surfaces (FCS). Among the individual surface categories, transport container lids, sink basins, cutting boards, food waste containers, handlers’ hands, and trays proved to be clear “hotspots,” where the odds of non-compliance were several-fold or even dozens of times higher compared to plates. Hygienic performance was consistently least favourable in summer, and the year-to-year analysis did not show a linear improving trend but identified a distinct peak non-compliance rate in 2023.

## 3. Discussion

### 3.1. Surface Microbial Load—Comparison with Other Studies

Our results confirm that in school catering, hygiene problems are not evenly distributed but appear in well-identifiable, concentrated “focal points”, including transport container lids, sink basins, and handlers’ hands. This picture aligns well with other domestic and international studies that identified the dishwashing environment, wet surfaces, and the human hand as among the most important risk factors [6,8,9].

The particularly concerning results for sink basins—where every third sample was above the limit—are consistent with studies describing standing water, areas around faucets, and drains as typical sites for biofilm formation and persistent contamination [23,24].

Plates, glasses, and utensils were much less frequently contaminated—which can likely be explained by the more standardized, better-controlled process of dishwasher cleaning—while areas involving manual dishwashing and hard-to-clean joints (silicone seals, lids, food waste containers) posed a greater risk.

Although the present work is a retrospective analysis of routine monitoring data (and thus does not quantify corrective actions), the identified hotspot surfaces suggest clear, practical targets for hygiene optimization. For sink basins and drains, emphasis should be placed on mechanical cleaning prior to disinfection, biofilm-prone zones (drains, faucet joints), and avoiding persistent standing water. For transport-container lids and seals, disassembly where possible, dedicated brushing of grooves, and verification of dishwasher/thermal steps may be warranted. For food waste containers, frequent emptying, drying, and strict separation from clean zones can reduce recontamination risk. Finally, the elevated counts on food handlers’ hands underscore the need for structured hand-hygiene training and periodic compliance verification [22].

Based on temporal trends, an improvement was observed during and immediately after the pandemic years, presumably due to the stricter hygiene measures introduced during the COVID-19 pandemic [7]. However, the deterioration of results seen in 2023 warns that maintaining hygiene standards requires continuous attention, and with the decrease in initial motivation, this better practice can easily revert [25,26].

### 3.2. Relationship Between Surface Microbiota and AMR

The structure and quantity of surface microbiota are closely related to the risk of resistance, as the resistome runs through the entire food chain: soil, feed, animals, slaughterhouses, and ready-to-eat foods all carry resistance genes [4]. The presence of AMR-burdened microbiota is also common in animal products and fresh vegetables, which come into direct contact with surfaces during kitchen preparation [3]. Seasonal studies of chicken and pork meats have also shown that the prevalence of antibiotic-resistant microorganisms and ciprofloxacin residues was higher in summer, which resonates well with the higher summer surface contamination observed in the present work [10]. These results suggest that a significant portion of the higher counts measured on kitchen surfaces may reflect a microflora in which resistant strains may also be present. Biofilms and standing water forming on surfaces can be resistance reservoirs. Biofilms forming in sink basins and drains are similar in structure to microbial communities in surface waters [27]. If resistant bacteria are introduced here via food or handlers’ hands, the biofilm can act as a long-term reservoir [28]. The high contamination of sink basins and food waste containers measured in the present study is thus also considered critical from an AMR perspective: these surfaces not only cause re-contamination but can indirectly contribute to the circulation of resistance genes towards wastewater and the environment. Frequent, sometimes sub-lethal concentrations of biocide exposure can increase the expression of efflux pumps, change cell membrane composition, and thereby enhance tolerance to antibiotics as well [12,13]. The investigation of biocide susceptibility and adaptation is complex, but increasing evidence supports that biofilms hardened by biocide exposure on surfaces can carry phenotypes that also provide protection against antibiotics [14]. Since in the present study one of the most critical surface groups is precisely the most heavily disinfected (sink, food waste container, transport container lids), there is a danger that improperly set disinfection protocols will, in the medium term, select a microbiota structure that is more resistant to both biocides and antibiotics.

The role of food handlers’ hands can be of paramount importance in the spread of resistant strains. Deficiencies in hand hygiene represent one of the most important pathways for pathogens—including antibiotic-resistant strains—to reach food and surfaces [29,30]. In the present study, the proportion of samples above the limit on handlers’ hands was 15%, which clearly indicates that personal hygiene is a weak point in school catering and is a risk factor in connection with AMR as well. The high microbial load of surfaces—especially in NFCS, wet, biocide-intensive zones—can be considered a potential reservoir and transmission node from an AMR perspective, through which resistant microorganisms can enter the school community.

### 3.3. Risk-Based Environmental Monitoring and Advanced Detection

Research discussing the application of risk-based environmental monitoring (EM) in the food industry emphasizes that monitoring programmes must focus on zones posing real risk, and formal compliance is not sufficient [23,27,31]. The results of the present study point in a similar direction: targeted monitoring of transport container lids, sinks, food waste containers, and handlers’ hands yields greater benefits than over-sampling already well-performing plates and glasses. Advanced microbiological detection methods are also important for future developments. With an approach based on live microbiome profiling, the examination of viable microbiota can indicate technological problems more sensitively than classical culture-based methods [32]. Regarding the future of microbiological risk assessment, metagenomics, targeted qPCR, and long-read sequencing are suitable methods for quantifying AMR genes occurring in food and may be suitable for characterizing the resistome of surfaces as well [11,33].

The practical lesson of the present study is thus twofold: on the one hand, rapid, indicator-based measurements (MicroTester) are very effective for tracking critical hygiene points on a large sample size; on the other hand, targeted molecular AMR monitoring would be needed on these highlighted surfaces (e.g., metagenomics, targeted ARG panel) for an AMR-focused step forward.

### 3.4. Protection of Children and Reduction in Antibiotic Use

A significant portion of childhood gastroenteritis is foodborne and often leads to medical care and antibiotic prescription—even if the pathogen is actually of viral origin [34]. Keeping surfaces clean and microbiologically controlled is thus indirectly a tool in the fight against AMR: fewer infections, less antibiotic use, slower spread of resistance. The practical implementation of the One Health approach happens through such relatively “simple” interventions: better hygiene in school kitchens, more conscious surface monitoring, more trained personnel, and more effective inspection [1,2,5]. These steps are simultaneously aligned with the objectives of the European Union’s Farm to Fork strategy, which handles the mitigation of AMR as a priority task alongside a sustainable and safe food supply [35].

### 3.5. Limitations and Future Research

The main limitations of the study include that only total mesophilic aerobic counts were measured, no detection of specific pathogens or resistance genes was performed, and we do not have direct data on the composition of the surface microbiome (e.g., the ratio of pathogenic and commensal, or resistant and susceptible microorganisms). In addition, the routine monitoring protocol did not include within-visit technical replicate swabs from the same surface spot; therefore, within-surface sampling variability and same-spot repeatability could not be quantified in this retrospective dataset. The study also covered only school catering kitchens, so the results can only be cautiously generalized to other forms of catering (hospitals, nursing homes, restaurants). At the same time, the large sample size, the multi-year time series, and the detailed breakdown by surface type significantly strengthen the reliability of the results.

In the future, it would be justified to conduct targeted AMR studies on critical surfaces (transport container lids, sinks, handlers’ hands) using culture-based and molecular methods; to map biocide use in detail (type, concentration, contact time), especially on NFCS; and to link surface microbiological results with disease data. Future prospective studies could also incorporate predefined replicate designs (e.g., adjacent standardized areas or other controlled approaches) specifically to estimate within-surface variability, alongside the validated analytical method.

## 4. Materials and Methods

### 4.1. Study Design and Sampling

**Figure 2:**
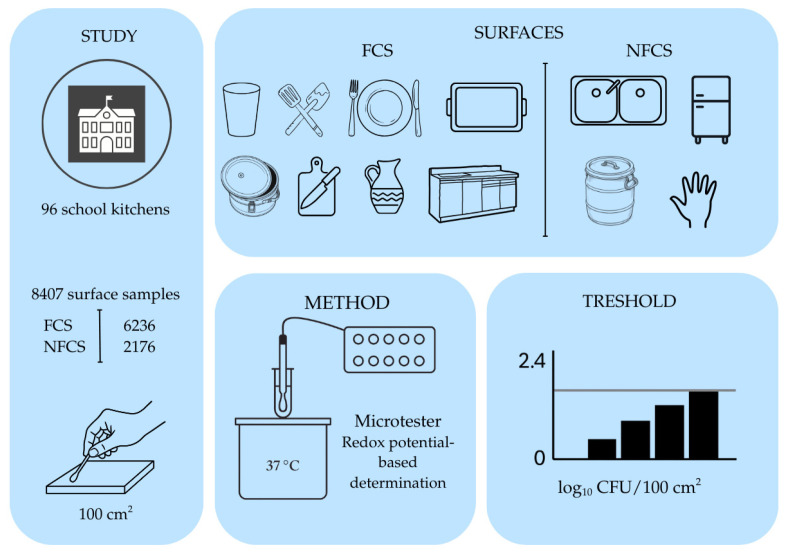
Schematic overview of study design, sampling strategy, and microbiological method.

The study took place between 2019 and 2024, involving 96 school catering kitchens. Sampling was conducted as part of routine hygiene monitoring. Sampling was performed by trained personnel from the department team at the University of Veterinary Medicine, Budapest, following a standardized written protocol. Kitchen staff were not involved in sample collection. Typically, up to two sampling rounds per kitchen per year were performed; however, the exact timing varied by kitchen and year, and sampling days were distributed throughout the calendar year. Therefore, seasonal analyses were based on the date of sampling, pooling observations across years. In each kitchen, samples were taken from identical, characteristic surface types according to a pre-fixed sampling plan (e.g., work tables, cutting boards, transport container lids, sink basins, hand wash basins, refrigerator handles, plates, glasses, knives, serving utensils). Surfaces were classified into food-contact (FCS) and non-food-contact (NFCS) groups. FCS included, among others, plates, glasses, utensils, trays, cutting boards, heat-retaining transport containers, jugs, and various plant tools (e.g., ladles, knives), while the NFCS category included internal surfaces of refrigerators, sink basins, handlers’ hands, and food waste containers. A total of 8412 surface samples were available. Typically, 8–10 different surfaces were examined on a single sampling day. On each sampling day, each selected surface item/area was sampled once; thus, each record in the dataset represents a single measurement per surface per visit. The same surface types could be re-sampled on later monitoring visits (up to two rounds per kitchen per year), but no same-day technical replicate swabs were collected from the same surface. For analytical comparisons, only surface types with at least 30 samples were included to ensure statistical comparability. Sampling was performed using sterile swabs, typically from a standardized surface of 100 cm^2^. After sampling, swabs were placed back into sterile transport tubes, kept refrigerated (4 ± 2 °C), and transported to the laboratory within 40 min. Upon arrival, samples were processed immediately. Sampling always took place on the morning following the previous day’s cleaning, before the opening of the kitchen; thus, the measured microbial load primarily reflected the effectiveness of the cleaning and disinfection practice (Figure 2).

### 4.2. Microbiological Analysis

Total mesophilic aerobic counts of the surfaces were determined using the MICROTESTER device (Micro Test Ltd., 1225 Budapest, Hungary) based on redox potential change. For the redox-potential measurement, each swab was aseptically placed into a MicroTester measuring cell containing 9 mL half-strength Tryptic Soy Broth (TSB), and the Time to Detection (TTD) was recorded at 30 °C. The essence of the method is that the redox potential of the medium decreases during the growth of microorganisms; detection time is inversely proportional to the initial count and can be converted to CFU/100 cm^2^ values based on standard curves [36]. Hungarian regulations prescribe a hygiene limit of 250 CFU/100 cm^2^ for food-contact surfaces; for comparability, the same threshold value was applied to non-food-contact surfaces as well. Raw CFU values were transformed to a log_10_ scale so that values of different magnitudes could be compared and extreme values would not distort averages. The 250 CFU/100 cm^2^ threshold corresponds to 2.4 log_10_ CFU/100 cm^2^.

### 4.3. Data Analysis

Data analysis was performed using IBM SPSS Statistics version 26 (IBM Corp., Armonk, NY, USA) software. The primary outcome variable was the binary hygiene status of the samples, based on the 250 CFU/100 cm^2^ threshold (0 = compliant, 1 = non-compliant). Counts were treated in log_10_ CFU/100 cm^2^ form. Continuous variables were described as mean ± standard deviation or median [interquartile range], and categorical variables as counts and percentages.

For inferential analyses, only surface categories with at least 30 samples were included, as odds ratios and confidence intervals derived from logistic regression become highly unstable at very low sample sizes. Surface types with fewer observations (77 samples in total, <1% of the dataset) were therefore reported descriptively only and were not entered into the regression models.

Differences in proportions between FCS and NFCS, as well as between individual surface types, were evaluated using the Chi-square test. Odds ratios (OR) and 95% confidence intervals associated with non-compliance were estimated using binary logistic regression; in the models, FCS/NFCS status, surface type (plate as reference), year, and season were considered as explanatory categorical variables.

To mitigate non-independence between samples, an outcome was also generated at the kitchen-day level (proportion of non-compliant surfaces, %), which was compared between seasons using one-way ANOVA, with a Tukey post hoc test if necessary. All tests were two-tailed, and the significance level was set at *p* < 0.05.

### 4.4. Data Availability

Raw monitoring records (sampling date, kitchen code, surface type, and measured microbial counts) were recorded electronically during routine hygiene monitoring and were archived in an institutional database maintained by the Department of Food Hygiene, Institute of Food Chain Science, University of Veterinary Medicine, Budapest. The anonymized dataset is available from the authors upon reasonable request.

## 5. Conclusions

Multi-year monitoring delivers an unambiguous message: acceptable average hygiene still leaves a recurring failure rate of ~12% of surfaces. Transport-container lids, sink basins, and food handlers’ hands repeatedly drive non-compliance and should be treated as primary control points. HACCP paperwork is not the limiting factor; consistent execution and verification of cleaning/disinfection and hand hygiene is. Given the potential role of environmental surfaces in maintaining and spreading AMR, closing these hygiene gaps is both a food-safety intervention and an AMR-control strategy. Monitoring should shift from broad, checklist-style sampling to risk-based surveillance of these hotspots and be complemented by periodic targeted pathogen/AMR testing. Continuous feedback, training, and protocol optimization are essential for sustained improvement in institutional catering within a One Health framework.

## Figures and Tables

**Table 1:** Surface-specific prevalence and odds of microbiological non-compliance.

No.	Surface Category	Total (N)	Non-Compliant (n)	Non-Compliant (%)	OR	95% CI	*p*-Value
1	Transport container lids	67	45	67.2	43.82	24.96–76.92	<0.001
2	Sink basins	878	288	32.8	10.46	7.93–13.79	<0.001
3	Cutting boards	102	22	21.6	5.89	3.90–7.82	<0.001
4	Food waste containers	366	75	20.5	5.52	3.47–9.99	<0.001
5	Food handlers’ hands	607	93	15.3	3.88	2.80–5.36	<0.001
6	Trays	708	97	13.7	3.40	2.47–4.68	<0.001
7	Refrigerators	883	100	11.3	2.74	1.99–3.75	<0.001
8	Work surfaces	937	97	10.4	2.47	1.80–3.40	<0.001
9	Eating utensils	858	72	8.4	1.96	1.40–2.75	<0.001
10	Jugs	31	2	6.5	1.48	0.35–6.31	0.598
11	Drinking cups/glasses	936	52	5.6	1.26	0.87–1.82	0.217
12	Kitchen equipment	365	17	4.7	1.05	0.61–1.80	0.869
13	Plates (reference)	1592	71	4.5	1.00	reference	—

Notes: Non-compliance was defined as a total aerobic mesophilic count > 250 CFU/100 cm^2^. Odds ratios (OR) were obtained from binary logistic regression with surface category as predictor; plates served as the reference category. Only surface categories with n ≥ 30 were included; rare categories (n < 30) are listed in Supplementary Table S1; 95% CI, *p*-values.

**Table 2:** Seasonal variation in the prevalence and odds of microbiological non-compliance (sample-level).

No.	Season	Total (N)	Non-Compliant (n)	Non-Compliant (%)	OR (vs. Summer)	95% CI	*p*-Value
1	Summer	1179	194	16.5	reference	-	-
2	Autumn	2693	375	13.9	0.82	0.68–0.99	0.041
3	Spring	2888	329	11.4	0.65	0.54–0.79	<0.001
4	Winter	1647	148	9.0	0.5	0.40–0.63	<0.001

Notes: Non-compliance was defined as >250 CFU/100 cm^2^. ORs were obtained from binary logistic regression with season as a categorical predictor; summer was the reference category.

**Table 3:** Seasonal variation in the daily proportion of non-compliant surfaces at the kitchen-day level.

Season	Mean Daily Non-Compliance (% ± SD)	ANOVA/Post Hoc Results
Spring	11.5 ± 13.5	Lower than summer (*p* = 0.008)
Summer	16.4 ± 17.4	Higher than spring (*p* = 0.008); Higher than winter (*p* < 0.001)
Autumn	14.0 ± 14.6	Higher than winter (*p* = 0.002)
Winter	9.1 ± 12.4	Lower than summer (*p* < 0.001); Lower than autumn (*p* = 0.002)

Notes: For each kitchen and sampling date, the proportion of non-compliant surfaces (%) was calculated and compared across seasons using one-way ANOVA (F(3, 876) = 7.85, *p* < 0.001; total kitchen–day observations n = 880). Post hoc comparisons were performed using Tukey’s test.

**Table 4:** Year-to-year variation in non-compliance and odds of non-compliance (sample-level).

Year	Total (N)	Non-Compliant (n)	Non-Compliant (%)	OR (vs. 2019)	95% CI	*p*-Value
2019	1378	184	13.4	1.00	reference	-
2020	841	106	12.6	0.94	0.72–1.21	0.61
2021	1976	152	7.7	0.54	0.43–0.68	<0.001
2022	1840	187	10.2	0.73	0.59–0.91	0.005
2023	1646	318	19.3	1.55	1.28–1.89	<0.001
2024	726	99	13.6	1.03	0.79–1.33	0.86

Notes: Non-compliance was defined as >250 CFU/100 cm^2^. ORs were obtained from binary logistic regression with the year as a categorical predictor; 2019 was the reference year.

## Data Availability

The raw data supporting the conclusions of this article will be made available by the authors on request—toth.andras.jozsef@univet.hu.

## Supplementary Materials

The following supporting information can be downloaded at: https://www.mdpi.com/article/10.3390/antibiotics15020120/s1, Table S1: Surface categories excluded from inferential analyses due to low sample size (n < 30).

## Abbreviations

The following abbreviations are used in this manuscript:

AMR	Antimicrobial Resistance
CFU	Colony Form Unit
NFCS	Non-Food Contact Surface
FCS	Food Contact Surface
HACCP	Hazard Analysis and Critical Control Points
GHP	Good Hygienic Practices
EM	Environmental Monitoring
