# Supplementary material for: Six-Year Environmental Surface Hygiene Monitoring in Hungarian School Kitchens (2019–2024): Hotspots, Seasonality, and One Health Implications
Source: Antibiotics (Basel). 2026 Jan 26;15(2):120. doi: 10.3390/antibiotics15020120 (PMC12937414; doi:10.3390/antibiotics15020120)
Supplement: Supplementary file 1 [file antibiotics-15-00120-s001.zip › antibiotics-4090169-supplementary.pdf]

Supplementary Table S1. Surface categories excluded from inferential analyses due to low sample size (n < 30).

No.	Surface category	Total samples (n)	Share of total dataset (%)*
1	Door handles	12	0.14
2	Serving carts	3	0.04
3	Hot holding unit	1	0.01
4	Plastic kitchen container	2	0.02
5	Shelves	7	0.08
6	Transport containers	8	0.10
7	Thermoboxes	24	0.29
8	Stoves	1	0.01
9	Boilers / cooking kettles	1	0.01
10	Kitchen containers	18	0.21

* Percentages are calculated relative to the full dataset of 8,412 samples. These categories (77 samples in total, ~0.9%) were reported descriptively only and were not included in logistic regression models.
